# Supplementary material for: Diagnostic yield, complications, pathology and anatomical features in CT-guided percutaneous needle biopsy of mediastinal tumours
Source: PLoS One. 2022 Nov 17;17(11):e0277200. doi: 10.1371/journal.pone.0277200 (PMC9671415; doi:10.1371/journal.pone.0277200)
Supplement: Attachment — Submitted filename: Resonse to Reviewers.docx [file pone.0277200.s001.docx]

**Dear Luka Brcic**

**We would like to thank the Editorial Board of PLOS ONE for considering our manuscript entitled “Diagnostic yield, complications, pathology and anatomical features in CT-guided percutaneous needle biopsy of mediastinal tumors” for publication.**

**Below we have carefully formulated our answers to the comments and questions of the reviewers. We have adjusted the manuscript following their helpful suggestions. The requested changes are highlighted in the revised version of the text using the Word “track changes” function.**

**We hope that our revision will facilitate the final Editorial decision.**

**…**

**Journal requirements:**

When submitting your revision, we need you to address these additional requirements.

1. Please ensure that your manuscript meets PLOS ONE's style requirements, including those for file naming. The PLOS ONE style templates can be found at

https://journals.plos.org/plosone/s/file?id=wjVg/PLOSOne_formatting_sample_main_body.pdf and

<https://journals.plos.org/plosone/s/file?id=ba62/PLOSOne_formatting_sample_title_authors_affiliations.pdf>.

Response: The manuscript and the title page has been changed accordingly.

2. Thank you for stating the following financial disclosure:

“NO”

At this time, please address the following queries:

a) Please clarify the sources of funding (financial or material support) for your study. List the grants or organizations that supported your study, including funding received from your institution.
b) State what role the funders took in the study. If the funders had no role in your study, please state: “The funders had no role in study design, data collection and analysis, decision to publish, or preparation of the manuscript.”
c) If any authors received a salary from any of your funders, please state which authors and which funders.
d) If you did not receive any funding for this study, please state: “The authors received no specific funding for this work.”

Please include your amended statements within your cover letter; we will change the online submission form on your behalf.

Response: The cover letter has been changed accordingly. We have added the following in the cover letter: “We did not receive any external funding for our study. We received internal research funding from the hospital. The hospital had no role in study design, data collection and analysis, decision to publish, or preparation of the manuscript. The authors received no specific funding for this work. The authors have declared that no competing interests exist.”

3. Thank you for stating the following in your manuscript:

“The study was funded by internal research funding from the hospital.”

We note that you have provided funding information that is not currently declared in your Funding Statement. However, funding information should not appear in the Acknowledgments section or other areas of your manuscript. We will only publish funding information present in the Funding Statement section of the online submission form.

Response: This has been deleted from the manuscript.

Please remove any funding-related text from the manuscript and let us know how you would like to update your Funding Statement. Currently, your Funding Statement reads as follows:

“No”

Please include your amended statements within your cover letter; we will change the online submission form on your behalf.

Response: Thank you for pointing this out. The cover letter has been changed accordingly.

4. Thank you for stating the following in your Competing Interests section:

“NO authors have competing interests”

Please complete your Competing Interests on the online submission form to state any Competing Interests. If you have no competing interests, please state ""The authors have declared that no competing interests exist."", as detailed online in our guide for authors at http://journals.plos.org/plosone/s/submit-now

 This information should be included in your cover letter; we will change the online submission form on your behalf.

Response: The cover letter has been changed accordingly.

5. PLOS requires an ORCID iD for the corresponding author in Editorial Manager on papers submitted after December 6th, 2016. Please ensure that you have an ORCID iD and that it is validated in Editorial Manager. To do this, go to ‘Update my Information’ (in the upper left-hand corner of the main menu), and click on the Fetch/Validate link next to the ORCID field. This will take you to the ORCID site and allow you to create a new iD or authenticate a pre-existing iD in Editorial Manager. Please see the following video for instructions on linking an ORCID iD to your Editorial Manager account: <https://www.youtube.com/watch?v=_xcclfuvtxQ>.

Response: Corresponding authors ORCID id is 0000-0003-3727-4649, this has been added.

6. Please amend your authorship list in your manuscript file to include list of authors.

Response: Thank you for pointing this out. We have added the list of authors to the manuscript.

7. We note that you have included the phrase “data not shown” in your manuscript. Unfortunately, this does not meet our data sharing requirements. PLOS does not permit references to inaccessible data. We require that authors provide all relevant data within the paper, Supporting Information files, or in an acceptable, public repository. Please add a citation to support this phrase or upload the data that corresponds with these findings to a stable repository (such as Figshare or Dryad) and provide and URLs, DOIs, or accession numbers that may be used to access these data. Or, if the data are not a core part of the research being presented in your study, we ask that you remove the phrase that refers to these data.

Response: We have now provided all data in the manuscript and removed the phrase “ data not shown”. Consequently we have now added the p-value in the manuscript for the previously not shown data.

Reviewers' comments:

Reviewer's Responses to Questions

**Comments to the Author**

1. Is the manuscript technically sound, and do the data support the conclusions?

The manuscript must describe a technically sound piece of scientific research with data that supports the conclusions. Experiments must have been conducted rigorously, with appropriate controls, replication, and sample sizes. The conclusions must be drawn appropriately based on the data presented.

Reviewer #1: No

Reviewer #2: Yes

2. Has the statistical analysis been performed appropriately and rigorously?

Reviewer #1: No

Reviewer #2: Yes

3. Have the authors made all data underlying the findings in their manuscript fully available?

The [PLOS Data policy](http://www.plosone.org/static/policies.action#sharing) requires authors to make all data underlying the findings described in their manuscript fully available without restriction, with rare exception (please refer to the Data Availability Statement in the manuscript PDF file). The data should be provided as part of the manuscript or its supporting information, or deposited to a public repository. For example, in addition to summary statistics, the data points behind means, medians and variance measures should be available. If there are restrictions on publicly sharing data—e.g. participant privacy or use of data from a third party—those must be specified.

Reviewer #1: Yes

Reviewer #2: Yes

4. Is the manuscript presented in an intelligible fashion and written in standard English?

PLOS ONE does not copyedit accepted manuscripts, so the language in submitted articles must be clear, correct, and unambiguous. Any typographical or grammatical errors should be corrected at revision, so please note any specific errors here.

Reviewer #1: Yes

Reviewer #2: Yes

5. Review Comments to the Author

Please use the space provided to explain your answers to the questions above. You may also include additional comments for the author, including concerns about dual publication, research ethics, or publication ethics. (Please upload your review as an attachment if it exceeds 20,000 characters)

Reviewer #1: Thank you for the opportunity to review the article entitled “Diagnostic yield, complications, pathology and anatomical features in CT-guided percutaneous needle biopsy of mediastinal tumors”. The authors retrospectively describe their experience using CT-guided needle biopsy to sample mediastinal tumors. While the data is interesting, there are major limitations to this study that needs to be clarified by the authors, some of which are inherent to the nature of the study. While the authors did mention the small number and retrospective nature of their study as a limitation further elaboration is required.

The authors rightfully describe other ways of sampling mediastinal tumors, such as endoscopic and surgical techniques. The authors should describe whether these techniques are used at their institution. As this is a retrospective study, it is important to have a sense of whether the percutaneous approach is used for all patients with mediastinal tumor or a carefully selected subset.

Response: The scope of our study was tumours selected for CT-guided biopsy only. We agree that this had to be clarified in the article.

For clarification, we have added a paragraph in Introduction:

The scope of this study are the tumours referred for CT-guided percutaneous biopsy. Endobronchial ultrasound (EBUS) can be used to obtain access to subcarinal, paratracheal, nd paraesophageal lymph nodes. [4]. At Akershus University hospital, mediastinoscopies is a rarely performed procedure and it has mostly been replaced by (EBUS) and CT guided biopsies. Mediastinoscopy requires general anesthesia and not all tumours of the mediastinum are accessible by this procedure [4]. CT guided percutaneous biopsy only require local anesthesia [7]. At Akershus University hospital, if a mediastinal tumour is inaccessible by EBUS, a CT guided percutaneous biopsy will be performed. CT guided procedure is also preferred over EBUS when there is a need for larger biopsy size which often is necessary to make tumour marker analysis.

Furthermore, we have rewritten a paragraph in Discussion as well as the following sentence accordingly to:

“The scope of this study are the tumors referred for CT-guided percutaneous biopsy. The tumours referred for CT-guided percutaneous biopsy is thoroughly selected. As mentioned, at Akershus University hospital tumours inaccessible by EBUS or tumours accessible by EBUS, but where there is need for a larger tissue sample, are referred for CT-guided percutaneous biopsy.”

Our data suggests that “when a tumour has been selected for CT-guided percutaneous needle biopsy,” the likelihood of a malignant diagnosis is higher, the further anteriorly the lesion is located within the mediastinum.

Along the same lines, most of the lesions sampled were in the inferior anterior mediastinum (30 lesions, %63 of procedures), with no cases in the inferior middle mediastinum. It would be helpful for the reader to know what the risk of complications with each compartment as the concern would be that the data may only reflect the safety of sampling lesions in the inferior anterior mediastinum rather than the whole mediastinum.

Response: Inferior middle mediastinum contains mainly the heart and we did not biopsy any tumours in the heart during this study. We have added an comprehensive new table (table 2) which presents number and type of complications within each compartment of anatomical mediastinum.

The authors should clearly state the risk of pneumothorax. The overall risk of pneumothorax was (25%, 12 cases) with intervention required in 4 cases (8%). Arguably, the risk of pneumothorax and the need for intervention should be calculated purely for the transpulmonary approach (60% of cases had pneumothorax, 20% required intervention)

Response: This has been changed accordingly.

Selection bias may explain why there were no mediastinal lesions sampled in the inferior middle mediastinum. Assuming that the institution has an endoscopy/bronchoscopy service, the lesions in the inferior middle mediastinum may have been sampled endoscopically given the proximity to major vascular structures. This again affects the way the results should be interpreted. Similarly, the recommendation to use compartment system vs the Felson system as result may not necessarily be accurate.

Response: Thank you for this comment. As mentioned above the scope of our study is mediastinal tumours selected for CT-guided biopsy. In the time period of the study, there were no lesions in the middle inferior mediastinum referred for CT-guided percutaneous biopsy since these often are reached using endoscopic technique.

I do not believe that the statement that the likelihood of malignant diagnosis is higher the further anteriorly the lesion is located in the mediastinum is valid unless all the mediastinal lesions at the institution are sampled via the percutaneous approach. Otherwise the conclusion is biased toward the lesions that were easier to access via the percutaneous approach compared to other approaches (endoscopic/Surgical)

Response: Thank you for the comment. The scope of our study is as mentioned percutaneous CT-guided biopsy. We agree that this had to be clarified in the article. The article has been changed accordingly. We have emphasized that our results may not necessarily be accurate for all mediastinal tumors but only those selected for CT guided biopsy.

The authors mention that two procedures were initially inconclusive and had to be re-biopsied. However, only one re-biopsy was included in the study. Can the authors explain why the other re-biopsy was not included (out of the study timeframe vs another sampling approach was used)?

Response: Thank you for the comment. The procedure was out of the timeframe of the study. A sentence has been added in paragraph nine in Results.

Minor comment
Introduction, 5th paragraph. As the authors are referencing more than 3 studies, would suggest changing the sentence to state that the complication rates range between 3-17%.

Response: We agree, this has been changed accordingly.

Reviewer #2: Dear Authors,

Thank You for an interesting article. The theme of this article is radiological, yet in the article, I do not find an explanation why is the CT imaging method of choice for percutaneous needle biopsy of this region and what are the advantages of this method in comparison with other imaging methods.

Response: Thank you for the comment. The reason why CT imaging method is preferred is explained in paragraph five in the Introduction.

In Figure 1. You show the needle tip in the lung window and the pathology in a mediastinal window. Is there any practical explanation for it (considering that the size of the lesion depending on the window can be underrated or overrated, the target components of the lesion during the biopsy are poorly discriminated in the lung window)?

Response:

Thank you for this interesting comment. We choose a mediastinal window setting during the biopsy because this gave us the best overview of the tumors located in the mediastinum. However, the window setting could easily be converted to lung window during the biopsy if we wanted to evaluate the lung parenchyma and determine if a pneumothorax had occurred.

How do You define the specific low-dose CT examination You used in the study?

Response: We used same low-dose CT that we use in the clinical setting to evaluate pneumothorax, lung hemorrhage or lung nodules. The protocol is without intravenous contrast. The effective dose per examination is about 1.5-2 mSv. This information has been added to the manuscript.

Postinterventional control radiography was performed. Is the sensitivity of this method in comparison to CT a limitation for the detection of postinterventional complications?

Response: Thank you for this comment. A low dose CT thorax scan was always performed directly after the procedure. To clarify we have moved the sentence regarding post interventional CT-control to the third paragraph under «CT-guided mediastinal biopsy procedure». After 2 hours, a frontal projection x-ray was performed. The sensitivity of chest x-ray is as mentioned lower compared to CT. However, in our experience most major complications occur right after ending the biopsy procedure. Therefore we conduct a low-dose CT primarily and a regular chest x ray 2 hours after. This is normal clinical procedure after chest intervention at our institution.

In which cases were chest drainage and surgical drainage performed?

Response: The following was added: "If a pneumothorax was symptomatic, if it evolved on the chest x-ray two hours after the procedure or if it occupied more than approximately 20-30% of the hemithorax” the radiologist performed a chest drainage using a pigtail catheter.

Regarding the diagnostic yield and adequate tissue material, did You find any statistical difference related to the tissue structure of the lesion (solid vs. cystic-solid)?

Response: We did not perform a statistical analysis related to tissue structure of the lesion. However, we did statistical analyses regarding heterogeneity, border regularity, calcification and volume. None of these features could distinguish whether the tumors were benign or malignant.

How was adequate tissue material defined?

Response: Thank you for this comment. Adequate tissue material was defined as enough material for the pathologists to make a diagnostic conclusion. This is now added in the result section.

In the discussion, much information pertains to results. Therefore, I would suggest rewriting the discussion and comparing more in detail the study results with current literature.
The text also needs some language adaptation and consistency in writing numbers (for instance - 2 or two).

Response: Thank you for your comments. We have rephrased and rewritten the discussion section.

6. PLOS authors have the option to publish the peer review history of their article ([what does this mean?](https://journals.plos.org/plosone/s/editorial-and-peer-review-process#loc-peer-review-history)). If published, this will include your full peer review and any attached files.


If you choose “no”, your identity will remain anonymous but your review may still be made public.


**Do you want your identity to be public for this peer review?** For information about this choice, including consent withdrawal, please see our [Privacy Policy](https://www.plos.org/privacy-policy).

Reviewer #1: **Yes:** Ala Eddin Sagar

Reviewer #2: No

[NOTE: If reviewer comments were submitted as an attachment file, they will be attached to this email and accessible via the submission site. Please log into your account, locate the manuscript record, and check for the action link "View Attachments". If this link does not appear, there are no attachment files.]

While revising your submission, please upload your figure files to the Preflight Analysis and Conversion Engine (PACE) digital diagnostic tool, <https://pacev2.apexcovantage.com/>. PACE helps ensure that figures meet PLOS requirements. To use PACE, you must first register as a user. Registration is free. Then, login and navigate to the UPLOAD tab, where you will find detailed instructions on how to use the tool. If you encounter any issues or have any questions when using PACE, please email PLOS at [figures@plos.org](mailto:figures@plos.org). Please note that Supporting Information files do not need this step.

*In compliance with data protection regulations, you may request that we remove your personal registration details at any time.* [*(Remove my information/details)*](https://www.editorialmanager.com/pone/login.asp?a=r)*. Please contact the publication office if you have any questions.*
